# Notes and Notices

**Published:** 1892-11

**Authors:** 


### NOTES AND NOTICES.

A Repertory to the Male Sexual Organs, compiled by Dr. F. G. Ohme, of Rosenburg, Oregon, appears in the October number of *The New York Medical Times* at page 197. It is valuable as far as it goes, and we cordially recommend it to our readers. The author might expand it to the size of a book and so add to the number of valuable monographs that have assisted homœopathic physicians in the difficult task of selecting the simillimum. Even the amount now published might, with propriety, be reprinted in pamphlet form for the convenience of practitioners, who would thus be able to make every-day use of it.

Childhood, a new magazine for parents about children, edited by Dr. Geo. Wm. Winterburn and Mrs. Florence Hull, the first number containing articles by Julian Hawthorne, Helen Campbell, Felix L. Oswald, Emma Marwedel, Rev. A. D. Mayo, Maria Louise Pool, Olive Thorne Miller, Mae St. John Bramhall, Moncure D. Conway, and other prominent writers will be issued November 25th. This magazine is intended to be a help and a guide to parents and teachers who desire to do the best possible for their children. It is *not* a *medical* magazine, but everything that concerns the welfare of the child will be duly considered. One dollar a year, ten cents a number. New York: A. L. Chatterton & Co., 78 Maiden Lane.

Our Dumb Animals, published at 19 Milk Street, Boston, by George T. Angell. Price, fifty cents a year. In the October number of this most useful publication is announced a number of prizes offered for the best essay on "The Effect of Humane Education in the Prevention of Crime." These prizes aggregate seven hundred dollars in cash. Send for sample copy.

Dr. R. B. Leach, of Paris, Texas, believes that the ravages of cholera can be prevented by the administration of Arsenic in the form of an inoculation; that it is in fact its vaccine. Accordingly he has written an open letter to the President of the United States urging upon his attention this plan of battling with cholera. He proposed to use ivory points charged with one-thirtieth of a grain of white Arsenic or hypodermic injections of from two to ten minims Fowler's or Pearson's Solution. This letter has been printed for public circulation.

Dr. T. F. Blanke has removed his residence to 2550 A, St. Louis Avenue, and his office to 2730 Jefferson Avenue, St. Louis, Missouri.

Dr. T. L. Hazard has removed from Anamosa, Iowa, to Iowa City, where he will take the practice of Dr. Cowperthwaite, who has located in Chicago.

Dr. Bender has removed his office and residence to Exeter Chambers, corner Exeter and Blagden Streets, next to Copley Square Hotel, Boston, Mass.

Dr. Joseph T. O'Connor, editor of *The North American Journal of Homœopathy,* has removed to No. 18 West 43d Street, New York.

